# Cloning of a novel tetrahydrofolate-dependent dicamba demethylase gene from dicamba-degrading consortium and characterization of the gene product

**DOI:** 10.3389/fmicb.2022.978577

**Published:** 2022-08-11

**Authors:** Na Li, Le Chen, E. Chen, Cansheng Yuan, Hao Zhang, Jian He

**Affiliations:** ^1^College of Life Science and Agricultural Engineering, Nanyang Normal University, Nanyang, China; ^2^Key Laboratory of Agricultural Environmental Microbiology, Ministry of Agriculture, College of Life Sciences, Nanjing Agricultural University, Nanjing, China; ^3^Jiangsu Academy of Agricultural Sciences, Institute of Germplasm Resources and Biotechnology, Nanjing, China; ^4^The Environmental Monitoring Center of Gansu Province, Lanzhou, China; ^5^College of Rural Revitalization, Jiangsu Open University, Nanjing, China

**Keywords:** dicamba, microbial consortium, gene clone, tetrahydrofolate-dependent demethylase, Dmt06

## Abstract

Dicamba, an important hormone-type systemic herbicide, is widely used to control more than 200 kinds of broadleaf weeds in agriculture. Due to its broad-spectrum, high efficiency and effectively killing glyphosate-resistant weeds, dicamba is considered as an excellent target herbicide for the engineering of herbicide-resistant crops. In this study, an efficient dicamba-degrading microbial consortium was enriched from soil collected from the outfall of a pesticide factory. The enriched consortium could almost completely degrade 500 mg/L of dicamba within 12 h of incubation. A novel tetrahydrofolate (THF)-dependent dicamba demethylase gene, named *dmt06,* was cloned from the total DNA of the enriched consortium. Dmt06 shared the highest identity (72.3%) with dicamba demethylase Dmt50 from *Rhizorhabdus dicambivorans* Ndbn-20. Dmt06 was expressed in *Escherichia coli* BL21 and purified to homogeneity using Co^2+^-charged nitrilotriacetic acid affinity chromatography. The purified Dmt06 catalyzed the transfer of methyl from dicamba to THF, generating the herbicidally inactive metabolite 3,6-dichlorosalicylate (3,6-DCSA) and 5-methyl-THF. The optimum pH and temperature for Dmt06 were detected to be 7.4 and 35°C, respectively. Under the optimal condition, the specific activity of Dmt06 reached 165 nmol/min/mg toward dicamba, which was much higher than that of Dmt and Dmt50. In conclusion, this study cloned a novel gene, *dmt06*, encoding an efficient THF-dependent dicamba demethylase, which was a good candidate for dicamba-resistant transgenic engineering.

## Introduction

Dicamba (3,6-dichloro-2-methoxybenzoate) is an important hormone-type systemic herbicide. It has been widely used to control more than 200 kinds of broadleaf weeds in the farmland of gramineous crops such as wheat, corn and rice (Stevens and Sumner, 1991; Huang et al., 2017). At present, the global annual usage of dicamba has reached 30,000 tons. Furthermore, the biotechnology giant Monsanto company has successfully developed transgenic soybean and cotton that were highly resistant to dicamba using the dicamba demethylase gene *DMO* (Behrens et al., 2007). These dicamba-resistant crops were approved by the US department of agriculture in 2015 and have been commercially planted since 2017 (Li et al., 2018). By 2019, the planting area of dicamba-resistant crops reached 50 million acres, accounting for 50% of the total planting area of soybeans in the United States.^1^ Therefore, it has important theoretical and application value to explore dicamba-degrading and detoxifying strain and gene resources (Barrows et al., 2014).

Microbial metabolism was the main factor in the dissipation of dicamba in the soil (Krueger et al., 1989; Taraban et al., 1993; Werwath et al., 1998). So far, many dicamba-degrading strains, such as *Stenotrophomonas maltophilia* DI-6, *Pseudomonas* sp. DI-8 (Krueger et al., 1989; Wang et al., 1997), *Sphingobium* sp. Ndbn-10, *Rhizorhabdus dicambivorans* (formerly *Sphingomonas* sp.) Ndbn-20, have been isolated (Yao et al., 2015). The initial degradation step of dicamba was demethylation in all of these isolates, generating the herbicidally inactive metabolite 3,6-dichlorosalicylate (3,6-DCSA; Krueger et al., 1989; Behrens et al., 2007; Yao et al., 2015). Two main types of dicamba demethylases have been identified: (1) monooxygenase type demethylase: Herman et al. (2005) identified a dicamba monooxygenase DMO from *S. maltophilia* DI-6, DMO was a three-component monooxygenase using NADH as the electron donor. (2) tetrahydrofolate (THF)-dependent methyltransferase: Yao et al. (2016) and Chen et al. (2019) identified two dicamba demethylases Dmt and Dmt50 from *R. dicambivorans* Ndbn-20. Both Dmt and Dmt50 were THF-dependent methyltransferases. They catalyzed the methyl transfer from dicamba to THF, generating 3,6-DCSA and 5-methyl-THF. The activities of Dmt and Dmt50 were severely inhibited by the product 5-methyl-THF, resulting in significantly lower demethylation effect than DMO, which limited their application values.

In this study, a highly efficient dicamba-degrading consortium was enriched from the soil collected from the outlet of a pesticide plant. The enriched consortium was able to almost completely degrade 500 mg/L of dicamba within 12 h of incubation. A THF-dependent dicamba demethylase gene, named *dmt06*, was cloned from the total DNA of the consortium. Dmt06 was heterogeneously expressed in *Escherichia coli* and purified by affinity chromatography. Furthermore, the enzymatic characteristics of Dmt06 was also investigated. Our results indicated that Dmt06 showed higher demethylation activity than previously reported THF-dependent demethylases Dmt and Dmt50 (Yao et al., 2016; Chen et al., 2019), indicating that the Dmt06 has good potential application in the construction of dicamba-resistant crops.

## Materials and methods

### Chemicals and media

Dicamba, 3,6-DCSA, NADH and THF were analytically pure. Methanol, acetonitrile, and acetic acid were chromatographically pure. All the chemicals were purchased from Sigma-Aldrich Company (Shanghai). Luria-Bertani (LB) broth and LB agar were obtained from Difco Laboratories (Detroit, MI). The minimal salt medium (MSM) consisted of the following components: 1.3 g K_2_HPO_4_, 0.86 g KH_2_PO_4_, 0.66 g (NH_4_)_2_SO_4_, 0.097 g MgSO_4_, 0.025 g MnSO_4_·H_2_O, 0.005 g FeSO_4_·7H_2_O, 0.0013 g CaSO_4_·6H_2_O per liter water, pH 7.0. For solid media, 15 g per liter of agar powder was added. All media were sterilized by autoclaving at 121°C for 20 min.

### Bacterial strains, plasmids, and primers

The *E. coli* strains and plasmids used in this study are listed in Table 1, and the primers used in this study are listed in Table 2. All *E. coli* strains were aerobically cultured in LB broth or agar at 37°C. Antibiotics were added at the following concentrations: kanamycin, 50 mg/L; ampicillin, 100 mg/L.

**Table 1:** Strains and vectors used in this study.

Strain or vector	Relative characteristics	Source or reference
*E. coli* DH5α	Host strain for cloning plasmid	TaKaRa
*E. coli* BL21 (DE3)	Host strain for expression vector	Vazyme Biotech
T vector	Clone vector; ampicillin^r^ (100 μg/ml)	TaKaRa
pET29a (+)	Expression vector; kanamycin^r^ (50 μg/ml)	Lab stock
pET29a*dmt06*	pET29a (+) derivative carrying *dmt06* gene	This study

**Table 2:** Primers used in this study.

Primer	DNA sequence (5′–3′)^a^	Purpose
27F	5´-AGAGTTTGATCCTGGCTCAG-3´	Amplification of the 16S rRNA gene of the dicamba degrader
1492R	5´-GGTTACCTTGTTACGACTT-3´	
pET-*dmt06*-F	TAAGAAGGAGATATACATATGGGAGAAGGACGGTCCCTTCA	Amplification of *dmt06* gene for expression in *E. coli* BL21
pET-*dmt06*-R	AGTGCGGCCGCAAGCTTCGGCGACCCGGCGGCCGTCGCC	
dF1	CTC(G)TTCG(A)ACCAGT(A)CC(G)CACCACATG	Amplification of the conserved regions of *dmt06* gene
dF2	TCGGCGACT(G)GC(G)ATCCTG(T)TAC(T)TA(G)	
dR1	GGC(A)TGGATC(G)C(G)CC(G)TACCCG(C)CTG(C)GCCG	
dR2	TC(G)AAGTTC(T)GAC(T)CAC(T)GACTTCATCGG	
uSP1	AGTACACGATGCAGTCGCCGACCACGT	Amplification of the upstream sequence of *dmt06*
uSP2 uSP3	AACCCGGCGAAGGTGTTGATGCCGA CTTTCAGGAACAGCTNNNNNNNNNGGTGGG	
dSP1	AGTGGAGCTGTCCGGCCCGTAC	Amplification of the downstream sequence of *dmt06*
dSP2	GACACCGTACGGTCGGCCATTCTC	
dSP3	AGAAGTACGGAATCGNNNNNNNNNGCACCC	

a The underlined bases indicate that they were overlapped and were used to construct plasmids by the In-Fusion.

### Enrichment of dicamba-degrading consortium

Soil sample used in this study was collected from the outfall of a pesticide factory. 10 g of the soil sample was transferred into a 250 ml Erlenmeyer flask containing 90 ml MSM supplemented with 500 mg/L dicamba. The Erlenmeyer flask was incubated at 30°C and 180 rpm on a shaker. At certain intervals, the remained dicamba concentration in the microbial consortium was determined using high-performance liquid chromatography (HPLC) as described below. When ~70% of the added dicamba was degraded, 10 ml of the enrichment culture was transferred into 90 ml fresh medium. The transfer was repeated for 5–6 times until the enriched consortium acquired high dicamba-degrading ability.

### Determination of the dicamba demethylase activity in the cell extract of the enriched consortium

To investigate which type of dicamba demethylase was in the enriched consortium. The bacterial cells of the final transferred consortium were collected by centrifugation at 6000 rpm for 10 min. After wishing twice with MSM, the cells were resuspended with ice-cold phosphate-buffered saline (PBS) buffer (50 mM, pH 7.4), and then disrupted by pulse sonication on ice with 15 s burst and 10 s pause for 15 min (Auto Science, UH-650B ultrasonic processor, 40% intensity), and the cell lysate was centrifuged at 12,000 rpm for 10 min at 4°C. The supernatant was collected as the cell extract. The protein concentration of the cell extract was quantified by the bicinchoninic acid (BCA) method using bovine serum albumin as the standard (BCA Protein Assay Kit, Sangon Biotech Shanghai Co., Ltd.). The demethylase activity of the cell extract toward dicamba was determined in a 300 μl mixture containing 100 mM PBS buffer, 2.0 mM NADH or 2.0 mM THF, 0.5 mM substrate, and 50 μl crude enzyme. The mixture was incubated for 5 min at 30°C, then the reaction was terminated by boiling at 100°C for 1.0 min. The conversion of substrate was analyzed by HPLC. One unit of dicamba demethylase activity was defined as the amount of enzyme that catalyzed the conversion of 1.0 nmol of dicamba per min.

### Cloning of the dicamba demethylase gene from the enriched consortium

The total DNA of the enriched consortium was extracted by Sodium dodecyl sulfate (SDS) high-salt method (Sambrook and Russell, 2001). To clone the dicamba demethylase gene from the total DNA of the enriched consortium, four degenerate primers including two forward primers and two reverse primers (Table 2) were designed according the conserved region of the reported THF-dependent demethylase gene sequences. These primers were paired for PCR amplification using the total DNA of the enrichment as a template. Amplification conditions: 94°C 3 min; 94°C 30 s, 55°C 30 s, 72°C 2 min, 33 cycles; 72°C 10 min. The acquired fragment was ligated into T-vector and transformed into *E. coli* DH5α. Sequencing of the fragment in the T-vector was performed using the ABI 3730xl DNA sequencer (Applied Biosystems). Then, the upstream sequence and downstream sequence of the acquired sequence were amplified by SEFA-PCR method, a DNA walking technology developed in our Lab (Wang et al., 2007), using the primers listed in Table 2.

### Expression of the dicamba demethylase gene and purification of the product

The dicamba demethylase gene was amplified by PCR using 2 × Phanta Master Mix (Vazyme Biotech Co., Ltd) using the primers listed in Table 2 and the total DNA extracted from the enrichment as the template. The PCR products were inserted into the NdeI-HindIII site of pET29(+) to generate the recombinant plasmid pET-*dmt06* using a one-step cloning kit (Vazyme Biotech). Then, pET-*dmt06* was transformed into *E. coli* BL21 (DE3) for expression. *Escherichia coli* BL21 (DE3) cells harboring pET-*dmt06* was grown in 100 ml of LB broth supplemented with 50 mg/L kanamycin at 37°C. When the absorbance at 600 nm reached 1.0, the cultures were induced with 0.4 mM isopropyl-*β*-D-thiogalactopyranoside (IPTG) for 10 h at 16°C. Then, cells were harvested by centrifugation at 12,000 rpm for 5 min. After washed twice with 100 mM PBS buffer (pH 7.4), the cells were resuspended in ice-cold PBS buffer, and then disrupted by sonication as described above, and the undisrupted cells and cell debris was removed by centrifugation at 12,000 rpm for 10 min at 4°C. The supernatant was charged onto 1-ml His-bind resin columns (HiTrap Talon crude; GE Healthcare Life Sciences), which had been activated by Co^2+^ and equilibrated with binding buffer. Following washing with binding/washing buffer containing different concentration of imidazole, the target protein was eluted with 5 ml of elution buffer [50 mM NaH_2_PO_4_, 300 mM NaCl, 100 mM imidazole (pH 8.0)]. The resultant fractions were dialyzed overnight at 4°C to remove imidazole in PBS buffer (100 mM, pH 7.4). The purities and molecular weights of the expressed protein was determined by 12% SDS-polyacrylamide gel electrophoresis (PAGE). The protein concentration of the cell extract was quantified by the BCA method. The dicamba demethylase activity was determined as described above. The metabolite was identified by mass spectrometry (MS) as described below.

### Study on the enzymatic characteristics

The temperature range was investigated at 50 mM PBS buffer (pH 7.4) under different temperatures (4°C–70°C), and the relative activity was calculated by assuming that the activity at 30°C was 100%. The pH range was investigated at pH values from 3.6 to 10.6 in three different buffering systems: 20 mM HAc-NaAc buffer (pH 3.6–5.8), 50 mM PBS buffer (pH 5.5–8.5), and 20 mM glycine-NaOH buffer (pH 8.6–10.6). The activity observed at pH 7.4 in PBS buffer was set as 100%, each value was the average from three independent experiments. For pH stability investigation, the enzyme was preincubated in buffers with different pH (pH 3.6–10.6) at 30°C for 4 h. Then, the remaining activity was assayed under the optimal condition. For thermostability investigation, the enzyme was preincubated in a water bath at different temperatures (30°C–70°C) for 120 min, and then the residual activity was assayed. To investigate the effects of potential inhibitors on demethylase activity, the enzyme mixture was preincubated for 30 min at 35°C, then the chemical agents (EDTA and SDS, final concentration of 5.0 mM) and metal ions (Li^+^, Na^+^, Mg^2+^, K^+^, Hg^2+^, Mn^2+^, Ni^2+^, Co^2+^, Zn^2+^, Cu^2+^, Ba^2+^, Al^3+^, Cd^2+^, Ag^+^, Fe^2+^, Fe^3+^, final concentration of 1.0 mM) were individually added, and the reactions were performed at 35°C for 30 min. Dicamba demethylase activity was assayed as described above and expressed as a percentage of the activity obtained without addition of the above compound.

### Chemical analysis

The collected samples were freeze-dried and dissolved in 1.0 ml of methanol. The solution was filtered through a 0.22 μm-pore-size Millipore membrane to remove particles. The concentrations of dicamba and metabolite were analyzed on an UltiMate 3,000 titanium system (Thermo Fisher Scientific) equipped with a C_18_ reversed-phase column (internal diameter, 4.6 mm; length, 250 mm; Agilent Technologies). The mobile phase was a mixture of ultrapure water (58.4%), acetonitrile (31.7%), methanol (7.5%), and 2.4% acetic acid (Yao et al., 2015). The flow rate was 1.0 ml min^−1^. A VWD-3100 single-wavelength detector was used to detect the UV absorption, the wavelengths for dicamba and 3,6-DCSA were 275 nm and 319 nm, respectively. The MS analysis was performed according to the method described by Liu et al. (2020).

Accession Number. The gene dmt06 sequence is deposited in the GenBank database under accession number ON828423.

## Results

### Enrichment of an efficient dicamba-degrading microbial consortium

**Figure 1:**
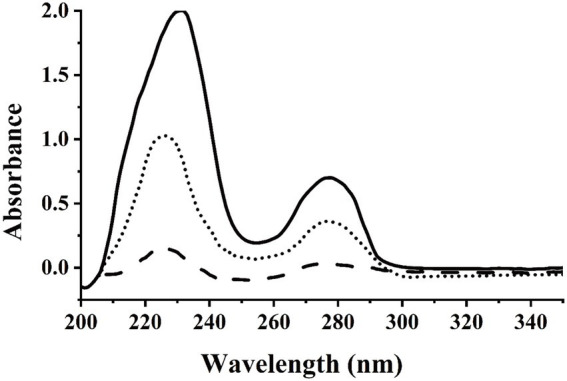
UV scanning detection of dicamba degradation by the enriched consortium. The sixth generation of the enriched consortium was inoculated into MSM supplemented with 500 mg/L of dicamba, which then was incubated in at 30°C and 180 rpm on a shaker. Solid line indicated the sample collected at 0 h, dotted line indicated the sample collected at 6 h, dashed line indicated the sample collected at 12 h.

**Figure 2:**
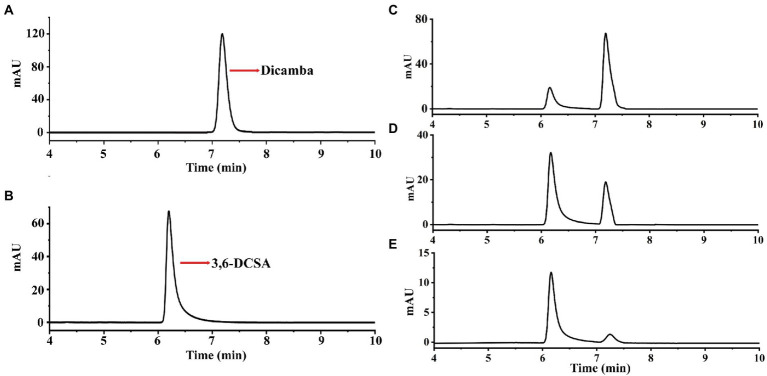
HPLC analysis of dicamba degradation by the enriched consortium. **(A)** The dicamba standard. **(B)** The 3,6-DCSA standard. **(C)** Sample collected at 4 h. **(D)** Sample collected at 8 h. **(E)** Sample collected at 12 h.

In this study, we used dicamba as a sole source of carbon to enrich dicamba-degrading microbial consortium. It took 11 days to degrade ~70% of the added 500 mg/L of dicamba for the first round of consortium. During the acclimation, the degradation ability of the consortium became stronger and stronger. After six rounds of transfer, the enriched consortium could almost completely degrade the added 500 mg/L of dicamba within 12 h incubation (Figure 1). HPLC analysis indicated that an intermediate metabolite was accumulated during the degradation, the retention time of the metabolite was equal to that of the 3,6-DCSA standard, and this metabolite disappeared with prolonged incubation (Figure 2). The results indicated that the enriched microbial consortium could efficiently degrade dicamba, and the first degradation step was demethylation to generate 3,6-DCSA, which could be further degraded by the enriched consortium.

### Degradation of dicamba by the crude enzyme of the enriched consortium

To date, two types of demethylases catalyzing the demethylation of methyl group-containing aromatics have been reported, one was the NADH-dependent monooxygenase, and another was the THF-dependent methyltransferase. In order to determine which type of dicamba demethylase was in the enrichment culture, the cell extract of the enriched consortium was obtained by ultrasonic disruption, and the dicamba demethylase activity in the cell extract was assayed in the presence of NADH and THF, respectively. The results showed that the cell extract added with THF acquired the dicamba demethylase activity, while the cell extract added with NADH could not convert dicamba (Table 3). The results suggested that the dicamba-degrading bacteria in the enriched consortium employed a THF-dependent demethylase to convert dicamba to 3,6-DCSA.

**Table 3:** Demethylase activities in cell extract of the enriched consortium.

Demethylase activity (nmol/min/mg)		
Addition of NADH	Addition of THF	No addition of NADH and THF
0	1.09 ± 0.23	0

### Cloning of the THF-dependent demethylase gene from the enriched consortium

**Figure 3:**
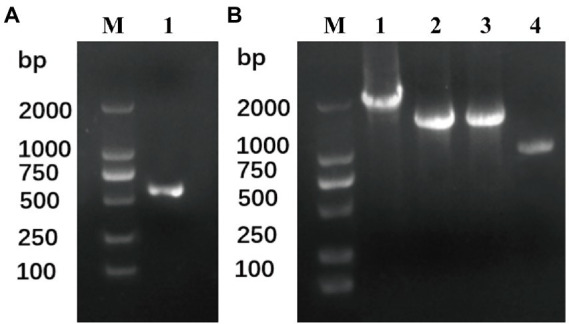
Amplification of the THF-dependent demethylase gene from the total DNA of the enriched consortium. **(A)** Fragment amplification from the total DNA of the enriched consortium using degenerate primers, Line M: DNA marker, line 1 amplified product using primer pair dF1 and dR1. **(B)** Amplified products from the upstream and downstream of the acquired fragment by SEFA-PCR, Line M: DNA marker, line 1: the first round SEFA-PCR from the fragment upstream, line 2: the first round SEFA-PCR from the fragment downstream, line 3: the second round SEFA-PCR from the fragment upstream, line 4: the second round SEFA-PCR from the fragment downstream.

To date, four THF-dependent demethylase genes have been reported: syringate demethylase DesA (Masai et al., 2004) and vanillate demethylase LigM (Abe et al., 2005) from *Sphingomonas paucimobilis* SYK-6, and dicamba demethylase Dmt (Yao et al., 2016) and Dmt50 (Chen et al., 2019) from *R. dicambivorans* Ndbn-20. To clone the dicamba demethylase gene, four degenerate primers (Table 2) including two forward primers (dF1 and dF2) and two reverse primers (dR1 and dR2) were designed according the conserved regions of the four THF-dependent demethylase genes, and then the primers were paired to amplify the dicamba demethylase gene from the total DNA of the enriched consortium. The results indicated that one pair of primers (dF1 and dR1) successfully amplified a fragment with clear band on gel electrophoresis (Figure 3A), the size of the acquired fragment was ~600 bp, which was consistent with the theoretical value. Sequencing results showed that the sequence of this fragment was homologous with 191–818 bp of *dmt50* with a similarity of 74.3%. Then, to clone the whole gene, primers of SEFA-PCR, were designed in the upstream and downstream of the acquired fragment. After two steps of SEFA-PCR amplification, clear bands with length of ~1–1.5 kb were obtained at both upstream and downstream of the acquired fragment (Figure 3B).

The acquired SEFA-PCR products were sequenced and finally assembled a complete gene, which named *dmt06* in this study. *dmt06* was 1,422 bp in size, encoding a 473 amino acid protein. Blast in GenBank of the NCBI indicated that Dmt06 was most related to some THF-dependent demethylases or aminomethyltransferase family protein. Interestingly, Dmt06 shared 100% identity with a putative aminomethyltransferase family protein from *Actinomadura parvosata* subsp. kistnae, and of the proteins with known function, Dmt06 showed the highest identity (72.3%) with dicamba demethylase Dmt50 from *R. dicambivorans* Ndbn-20, and shared 46.2% identity with Dmt.

### Expression and purification of Dmt06

**Figure 4:**
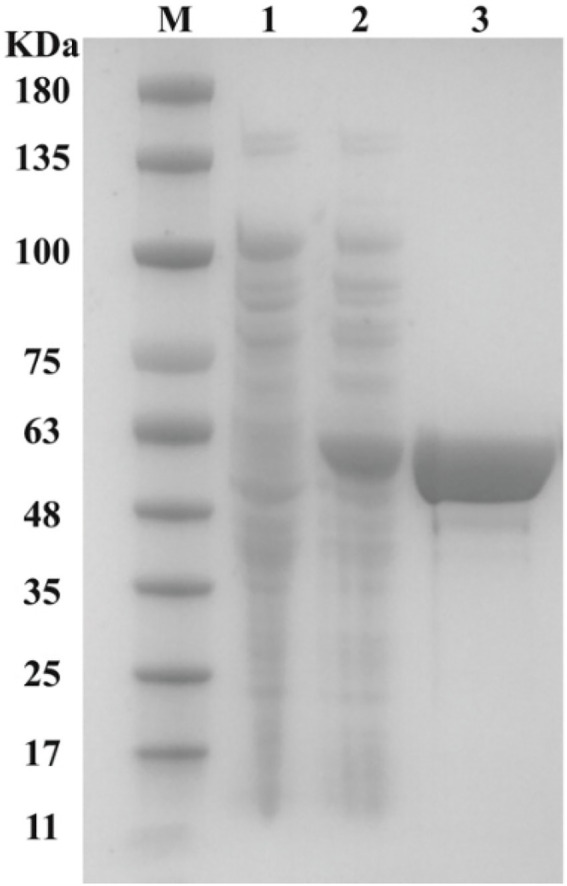
SDS-PAGE analysis of the purified Dmt06. Lane M: protein molecular marker; lane 1: crude extract of *E. coli* BL21 harboring pET29a; lane 2: crude extract of *E. coli* BL21 harboring pET-*dmt06*; lane 3: purified Dmt06.

To investigate the function of Dmt06, the *dmt06* gene was ligated into plasmid pET29a(+) and expressed in *E. coli* BL21(DE3) under the induction of IPTG. Then, the recombinant Dmt06 was purified to homogeneity using Co^2+^-charged nitrilotriacetic acid affinity chromatography (Figure 4). SDS-PAGE analysis indicated that the molecular mass of the denatured protein was ~55 kDa, which was consistent with the theoretical mass of the tagged protein (52.3 kDa). Dmt06 was stored at −80°C in 100 mM PBS buffer (pH 7.4) with 10% glycerol and 0.3 mM EDTA, 90% of its activity was retained when stored for 2 months, indicating that Dmt06 was very stable.

**Figure 5:**
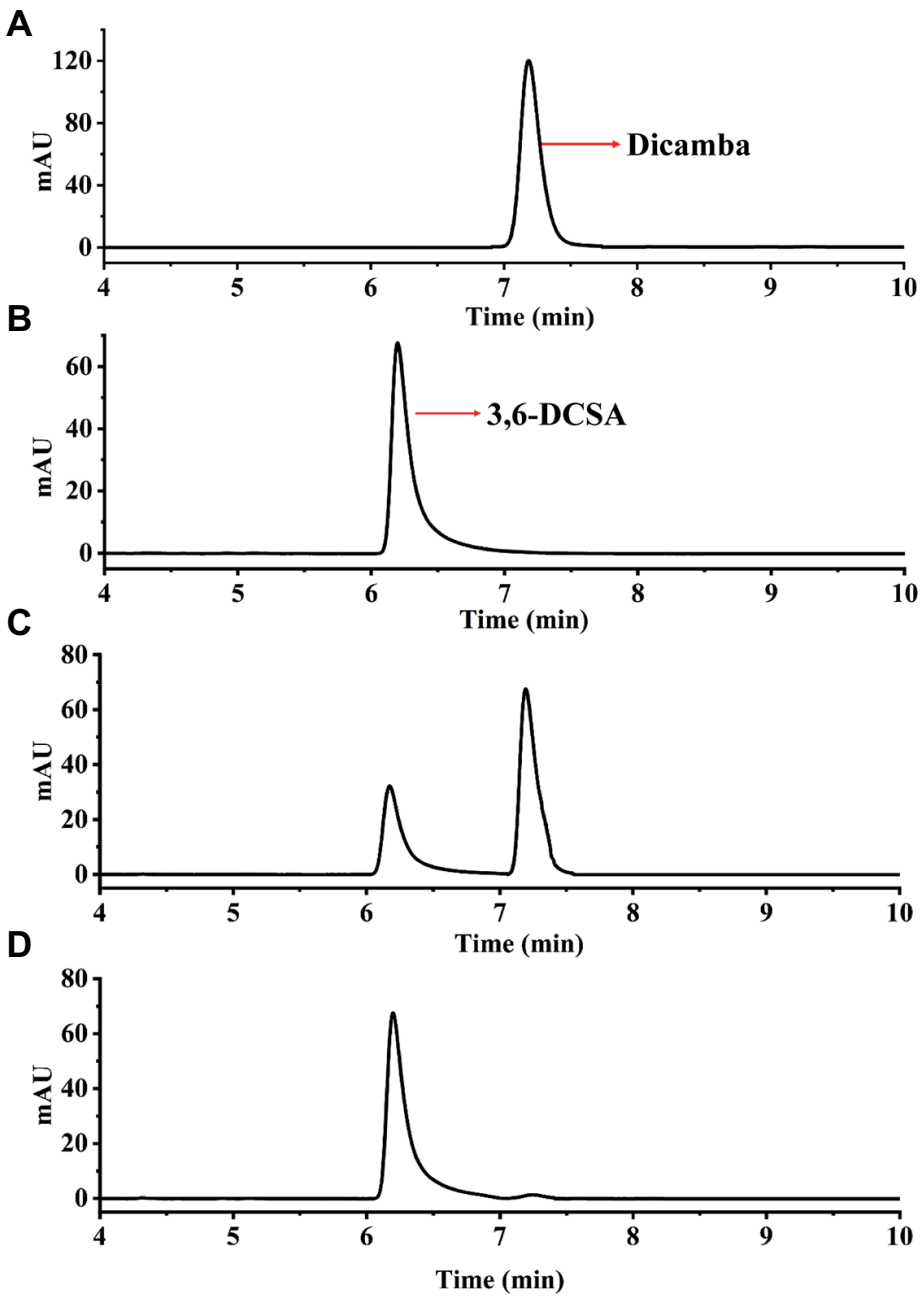
HPLC analysis of the product produced during dicamba conversion by Dmt06. **(A)** The dicamba standard. **(B)** The 3,6-DCSA standard. **(C)** Sample collected at 5 min. **(D)** Sample collected at 10 min.

**Figure 6:**
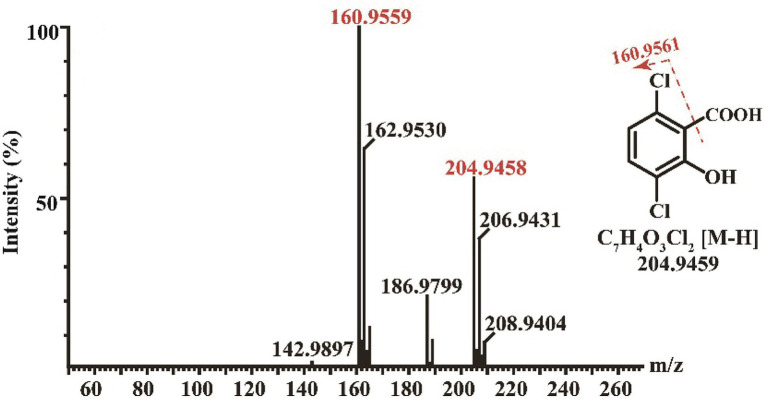
MS analysis of the product produced during dicamba conversion by Dmt06.

Enzymatic assays showed that Dmt06 could transfer dicamba to a product with a retention time at 6.25 min, which was equal to that of the 3,6-DCSA standard (Figure 5). MS analysis showed that the product had a prominent deprotonated molecular ion peak at m/z 204.95 (M-H)^−^ with a fragment peak at m/z 106.95 (Figure 6), this ion peak characteristic was also consistent with that of 3,6-DCSA. Thus, the product was identified as 3,6-DCSA. The above results demonstrated that Dmt06 was a demethylase catalyzing the conversion of dicamba to 3,6-DCSA.

### Enzymatic characteristics of the purified Dmt06

The purified Dmt06 displayed dicamba demethylase activity only in the presence of THF, indicating that Dmt06 was a THF-dependent demethylase. The activity of Dmt06 was detected from 10°C to 60°C and at pH ranging from 5.5 to 10.0. The optimum pH and temperature for Dmt06 was 7.4 and 35°C, respectively (Supplementary Figures S1, S2). In the stability studies, more than 70% of the activity of Dmt06 was retained after incubation at 50°C for 120 min, and <20% of its activity was retained after incubation at 60°C for 120 min. The results indicated that Dmt06 was stable at 50°C, but become unstable when temperature raised to 60°C. The effects of various metal ions and potential inhibitors on the activity of Dmt06 are shown in Supplementary Figure S3. The activity of Dmt06 was severely inhibited by 1.0 mM of Hg^2+^, Co^2+^, Zn^2+^, Cd^2+^, Ag^2+^, and 5.0 mM of SDS, and was moderately inhibited by 1.0 mM of Mn^2+^, Ni^2+^, Cu^2+^, Ba^2+^, and Al^3+^. On the other hand, 1.0 mM of Li^+^, Na^+^, K^+^, Mg^2+^, Ca^2+^, Fe^2+^, Fe^3+^, and 5.0 mM EDTA had no obviously effect on the activity of Dmt06. At the optimal condition, the incubation of 0.1 mg of Dmt06 for 5 min resulted in a specific activity of 165 nmol/min/mg toward dicamba. Dmt06 could not catalyze the methyl transfer of vanillate, syringate, isoproturon, and alachlor, indicating that Dmt06 possessed a very narrow substrate spectrum.

## Discussion

The planting of glyphosate-resistant transgenic crops combined with the application of glyphosate can effectively kill weeds without damaging the crops, thus proving an efficient and low-cost weed control strategy (Barrows et al., 2014). Therefore, the planting area of glyphosate-resistant GM crops have been rapidly expanded since their commercialization in the early 1990s, reaching ~150 million hectares in 2018 (See footnote 1). However, long-term high-intensive use of glyphosate has resulted in increasing resistance of weeds (Cerdeira and Duke, 2006). It was reported that at least 41 kinds of weeds in 29 countries have developed strong resistance to glyphosate, which makes the weeding strategy of glyphosate-resistant transgenic crop ineffective. Therefore, it is necessary to screen new target herbicides and construct corresponding transgenic crops. Dicamba is a broad-spectrum herbicide that can effectively kill glyphosate-resistant weeds. In particular, dicamba has been commercially used for more than 60 years since it was developed in the 1960s (Tomlin, 2006), but so far, few weeds have developed resistance to dicamba. Therefore, dicamba is an ideal target herbicide for the next generation of herbicide-resistant engineering, in which the dicamba detoxification genes have great application potential.

Previous studies have indicated that dicamba could be degraded by soil bacteria, and the initial step of microbial degradation was demethylation to generate 3,6-DCSA, which was herbicidally inactive (Li et al., 2018). Thus, dicamba demethylase gene is an ideal herbicide-resistant gene for herbicide-resistant engineering. So far, a NADH-dependent dicamba monooxygenase DMO and two THF-dependent dicamba demethylases Dmt and Dmt50 have been identified. Biological giant Monsanto has successfully used the oxygenase gene of *DMO* to construct dicamba-resistant soybean and corn, which have been commercially planted on a large scale. Compared with DMO, the advantage of the THF-dependent demethylases Dmt and Dmt50 is that they do not need reducing force NADH, so it is more energy-saving. In addition, the 5-methyl-THF generated during dicamba demethylation can be used for the synthesis of purine, pyrimidine, glycine and methionine, as well as the methylation of DNA, fatty acids and enzymes (Sullivan et al., 2021; Menezo et al., 2022). However, the disadvantage of Dmt and Dmt50 is that their activities were lower than that of DMO, which greatly limits their abilities to detoxify dicamba.

In this study, we enriched a highly efficient dicamba-degrading microbial consortium, the sixth generation of the enrichment could almost completely degrade 500 mg/L dicamba within 12 h incubation. In previous reports, *Sphingobium* sp. Ndbn-10 and *R. dicambivorans* Ndbn-20 degraded 2.25 mM (~ 497 mg/L) of dicamba within 36 h and 72 h, respectively, and *Pseudomonas maltophilia* DI-6 degraded 97% of 1,000 mg/L dicamba within 30 h (Krueger et al., 1989; Yao et al., 2016). Thus, the enriched consortium had a relatively high dicamba-degrading efficiency when compared with previous studies. To isolate pure strains that capable of degrading dicamba, the enriched consortium was serially diluted, the diluent was spread on LB plate and incubated at 30°C for 5 days. Colonies with different morphology were selected to test their dicamba-degrading abilities. We selected ~100 colonies, unexpectedly, none of them could degrade dicamba. The possible reason might be that the bacteria responsible for dicamba degradation in the enriched consortium were unculturable. Activities study of the crude extract of the consortium indicated that the degradation of dicamba was initiated by demethylation, which catalyzed by an unknown THF-dependent demethylase (named Dmt06 in this study). To clone the gene, a fragment of *dmt06* was firstly successfully amplified from the total DNA of the enriched consortium by PCR using two pairs of degenerate primers, which designed according to the conserved region of reported THF-dependent demethylases, and then the full length of *dmt06* was obtained by DNA walking using SEFA-PCR method. Interestingly, results of blastp in the Non-redundant protein sequences (nr) database of NCBI showed that Dmt06 shared 100% identities with a putative aminomethyltransferase family protein from *Actinomadura parvosata* subsp. kistnae, and ~70%–98% identities with a lot of putative aminomethyltransferase family proteins from indigenous soil bacteria *Rhizobiales*, *Nonomuraea*, *Rhizorhabdus*, *Proteobacteria*, *Rhizobium*, *Mesorhizobium*, *Sinorhizobium*, *Tianweitania*, and *Microbacterium*. The wide distribution of this gene in soil bacteria suggested that it may have important functions, e.g., it is possible that the gene participates in the demethylation process of some natural occurred methyl-containing aromatic compounds. Results of blastp in the UniProtKB/Swiss-Prot (swissprot) database of NCBI indicated that Dmt06 was most related to the two reported THF-dependent demethylases Dmt and Dmt50. However, the identities between them were <75%, these analyses suggested that *dmt06* might encode a novel THF-dependent methyltransferase gene that differs from *dmt* and *dmt50*. Furthermore, Dmt06 was also obviously different from Dmt and Dmt50 in size and enzymatic characteristics. E.g., Dmt06 has 473 amino acids, while Dmt and Dmt50 have 466 and 475 amino acids, respectively. Dmt06 had a relatively narrow pH range (5.5–10.0) than that of Dmt (5.0–10.0) and Dmt50 (3.6–10.0), Dmt06 could tolerate 50°C, which was higher than that of Dmt (45°C) but much lower than that of Dmt50 (75°C). The optimal pH and temperature for Dmt06 (7.4 and 35°C, respectively) were also different from that of Dmt (8.0 and 30°C, respectively) and Dmt50 (8.0 and 45°C, respectively). Most important, the specific activity of Dmt06 reached 165 nmol/min/mg toward dicamba, which was significantly higher than that of Dmt (114 nmol/min/mg; Yao et al., 2016) and Dmt50 (146 nmol/min/mg; Chen et al., 2019), indicating that Dmt06 has stronger dicamba detoxification ability than Dmt and Dmt50. Thus, *dmt06* is a potential candidate for the engineering of dicamba-resistant transgenic crops and bioremediation of dicamba residue pollution in environment. At the same time, the sequence comparison and structure analysis of Dmt06, Dmt and Dmt50 can provide a basis to elucidate the catalytic mechanism of the THF-dependent demethylase. In the future, we will study the structure and the key active sites of this new dicamba demethylase, and improve the activity of Dmt06 through directed evolution technology and rational protein design.

## Conclusion

In conclusion, this study cloned a novel THF-dependent dicamba demethylase gene *dmt06* from an efficient dicamba-degrading microbial consortium. Dmt06 were synthesized in *E. coli* BL21 and purified as His-tagged enzymes. Enzymatic assay showed that the dicamba demethylation activity of Dmt06 was much higher than that of Dmt and Dmt50, indicating that it has good application value in the dicamba-resistant transgenic engineering.

## Data availability statement

The data presented in the study are deposited in the NCBI GenBank repository, accession number ON828423.

## Author contributions

NL and LC conceived the presented idea. NL and JH contributed to the writing and prepared the figures and tables. EC, CY and HZ participated in revising the manuscript. All authors contributed to the article and approved the submitted version.

## Funding

This work was financially supported by the National Natural Science Foundation of China (no. 31900082); the China Postdoctoral Science Foundation (no. 2021M701735); and the High-qualified Talents Scientific Research Startup Foundation of Nanyang Normal University (2019ZX013).

## Conflict of interest

The authors declare that the research was conducted in the absence of any commercial or financial relationships that could be construed as a potential conflict of interest.

## Publisher’s note

All claims expressed in this article are solely those of the authors and do not necessarily represent those of their affiliated organizations, or those of the publisher, the editors and the reviewers. Any product that may be evaluated in this article, or claim that may be made by its manufacturer, is not guaranteed or endorsed by the publisher.

## Supplementary material

The Supplementary material for this article can be found online at: https://www.frontiersin.org/articles/10.3389/fmicb.2022.978577/full#supplementary-material

## Footnotes

^1^ http://www.isaaa.org
