# Supplementary material for: Cloning of a novel tetrahydrofolate-dependent dicamba demethylase gene from dicamba-degrading consortium and characterization of the gene product
Source: Front Microbiol. 2022 Aug 11;13:978577. doi: 10.3389/fmicb.2022.978577 (PMC9404685; doi:10.3389/fmicb.2022.978577)
Supplement: Supplementary file 1 [file Data_Sheet_1.PDF]

Running title: Dicamba Demethylase Gene *dmt06*

Cloning of A Novel Tetrahydrofolate-Dependent Dicamba Demethylase Gene from Dicamba-degrading Consortium and Characterization of the Gene Product

Na Li[1, 5], Le Chen[2], E Chen[3], Cansheng Yuan[4], Hao Zhang[1], Jian He[4, 5*]

[1] College of Life Science and Agricultural Engineering, Nanyang Normal University, Nanyang, China

[2] Institute of Germplasm Resources and Biotechnology, Jiangsu Academy of Agricultural Sciences, Nanjing, China

[3] The Environmental Monitoring Center of Gansu Province, Lanzhou, China

[4] College of Rural Revitalization, Jiangsu Open University, Nanjing, China

[5] Key Laboratory of Agricultural Environmental Microbiology, Ministry of Agriculture, College of Life Sciences, Nanjing Agricultural University, Nanjing, China

[*]Corresponding author

E-mail address: 530241892@qq.com; hejian@njau.edu.cn (Jian He)

Tel: +86-25-84396685; Fax: +86-25-84395326

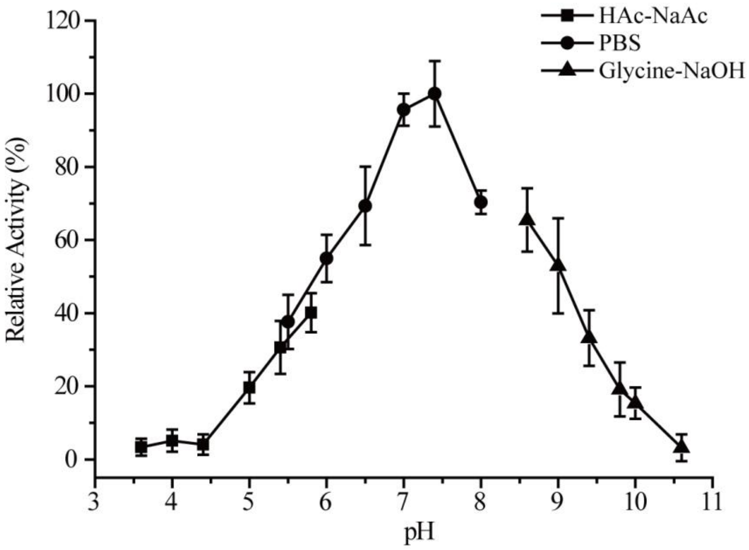

Figure S1. Effects of pH on the activities of Dmt06. Three buffering systems : ■ 20 mM HAc-NaAc buffer (pH 3.6 to 5.8), ● 50 mM PBS buffer (pH 5.5 to 8.5) and ▲ 20 mM glycine-NaOH buffer (pH 8.6 to 10.6). The relative activity was calculated by assuming that the activity at pH 7.4 in PBS buffer was 100%.

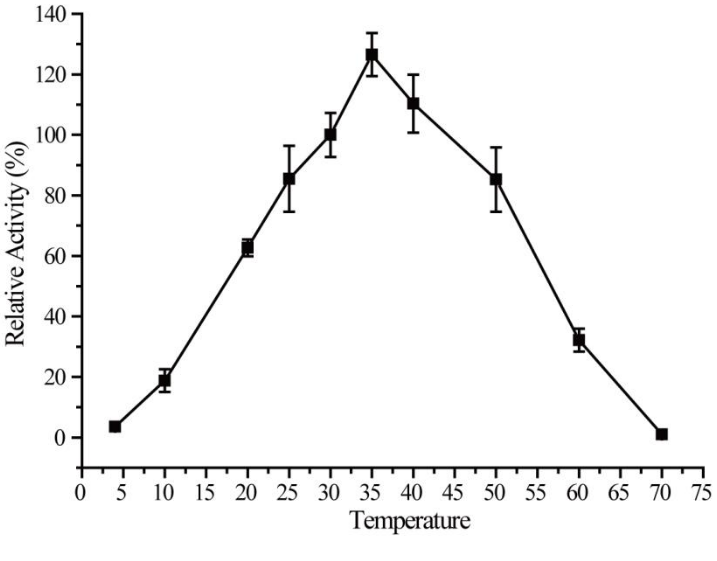

Figure S2. Effects of temperature on the activities of Dmt06. Dmt06 was incubated in 50 mM PBS buffer (pH 7.4) at different temperatures (4-70°C) for 30 min, and the relative activity was calculated by assuming that the activity observed at 30°C was 100%.

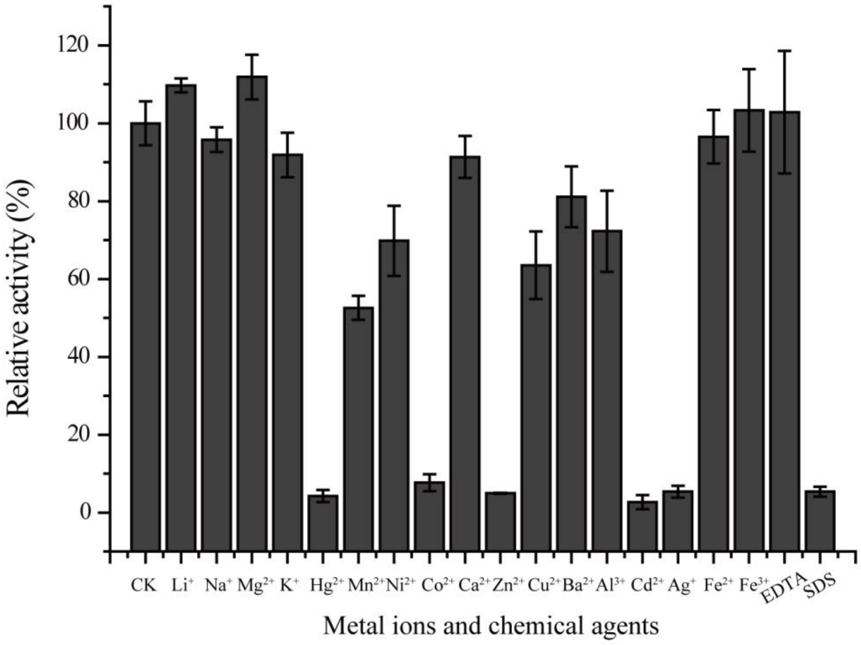

Figure S3. Effects of metal ions and chemical agents on the activities of Dmt06. CK: control without addition of any metal ions or chemical agents, and its relative activity was defined as 100%. The concentration of each ion was 1.0 mM, and the concentrations of EDTA and SDS were 5.0 mM.

The nucleotide sequence of *dmt06*:

ATGGGAGAAGGACGGTCCCTTCAGGACCTGATCGACGCGACGCCGAACCT
GGTGGATTACTTCCACAACGACACCATCGCCCCGCACTACCGGGCGCGTAC
GAGCCTGACCTCGGCGTTCGTCCCGCCGGAATTCTCCAACTGGCGGGATGA
GCAGCGGGCCTGGCGCGAGAGCGTGATCCTGTTCGACCAGTCCCACCACA
TGCCCGAGCTGTTCCTGAAAGGCCCGGACGCGCTGCGCCTGCTGACGCGG
GTCGGCATCAACACCTTCGCCGGGTTCGGCCCGGGCCGGGCCAAGCAGCT
CGTCGCGTGCACGCCCCGCGGGCACGTGGTCGGCGACTGCATCGTGTACT

GCCTCGGCGCCGACAGCTTCGAGCTCGTCAGCGGCATGGCGGTGCTGAAC
TGGGTGCACTACCAGGCGGAGACGGGCGGATACGACGTCACGATCGAGCG
GGACGCGCCGACTCCGTACAACCCGGCGGGCAGGCGCGTCTTCTACCGTT
TCCAGCTCGACGGGCCGCACGCGGGCAAGACCTTCGGCGACGCCGTCGAA
GGGCCGGTGCCGGACATCCCGTTCTTCAGGACCGCGCGGGTGCGGATCGG
CGGAGCCGAGGTCCTGGCCCTGCGCCACGGAATGGCCGGCCACCAGGGAG
TGGAGCTGTCCGGCCCGTACGAGGAGCTCGACACCGTACGGTCGGCCATT
CTCGCGGCCGGCGAGAAGTACGGAATCGTCCAGGGCGGCACCCAGTCGTA
TTTCAGCACCATCTTCGAGAGCGGCTGGATCGCCTACCCGCTGGCCGGGAT
CTACACCGGCGAGGAGCTGAGGCACTTCAGGCAGTGGCTGCCGGCGACCG
GCTGGGAGGCCAACGCACAACTCGGCGGCAGCTTCGTCTCGGCCGACATC
GAGGACTACTACGTCACCCCGTGGGATCTGGGTTATGACCGCCTGCTCAAG
TTCGACCACGACTTCATCGGCCGCCCGGCGCTCGAGAGCCTGGCCTCCCG
GCCGCACCGCAGGAAGGTGACCCTCGTCTGGAACGAGGAGGACGTGCTG
CGGATCCTGGCCTCGCAGTTCGGGACGGGCCCCCGGTTCAAGTCGCTGGA
CTTCCCGGTCTCGTTCTACGGCTTCCCCCAGTTCGACGAGGTCCGCGACGA
GGCCGGCGGGATGGCGGGACTGTCCAGCCACTGCGGCTACAGCAACAACG
AGGGCGCCGTGCTCTCGCTGGCCATGCTCAACGAGCGGCACGCGACTCCT
GGAACGCAGGTCGTGCTCGTCTGGGGCGAGCCGGACGGTGGCTCCAGAA
AGCCCCACGTGGAGCGCCACCACCAGACGACCGTCCGCGCCACCGTCGCG
CCGGCACCGTACGCCCGGCACGTCCGCGAGGTGAAACGGGCGACGATGAC
GGCCGCCGGGTCGCCGTGA

The amino acid sequence of Dmt06:

MGEGRSLQDLIDATPNLVDYFHNDTIAPHYRARTSLTSAFVPPEFSNWRDEQRAWRESVILFDQSHHMPELFLKGPDALRLLTRVGINTFAGFGPGRAKQLVACTPRGHVVGDCIVYCLGADSFELVSGMAVLNWVHYQAETGGYDVTIERDAPTPYNPAGRRVFYRFQLDGPHAGKTFGDAVEGPVPDIPFFRTARVRIGGAEVLALRHGMAGHQGVELSGPYEELDTVRSAILAAGEKYGIVQGGTQSYFSTIFESGWIAYPLAGIYTGEELRHFRQWLPATGWEANAQLGGSFVSADIEDYYVTPWDLGYDRLLKFDHDFIGRPALESLASRPHRRKVTLVWNEEDVLRILASQFGTGPRFKSLDFPVSFYGFPQFDEVRDEAGGMAGLSSHCGYSNNEGAVLSLAMLNERHATPGTQVVLVWGEPDGGSRKPHVERHHQTTVRATVAPAPYARHVREVKRATMTAAGSP
